# Ethnic Variability in Glycemic Response to Sucrose and Isomaltulose

**DOI:** 10.3390/nu9040347

**Published:** 2017-04-01

**Authors:** Wei Shuan Kimberly Tan, Sze-Yen Tan, Christiani Jeyakumar Henry

**Affiliations:** 1 Clinical Nutrition Research Centre (CNRC), Singapore Institute for Clinical Sciences (SICS), Agency for Science, Technology and Research (A*STAR) and National University Health System, Centre for Translational Medicine, 14 Medical Drive #07-02, MD 6 Building, Yong Loo Lin School of Medicine, Singapore 117599, Singapore; kimberly_tan@sics.a-star.edu.sg (W.S.K.T.); Tan_Sze_Yen@sics.a-star.edu.sg (S.-Y.T.); 2 Department of Biochemistry, National University of Singapore, 8 Medical Drive, Singapore 117596, Singapore

**Keywords:** sucrose, isomaltulose, ethnic differences, glycemic response

## Abstract

The aim of this study was to compare the glycemic response of Caucasians and Asians to two disaccharides of different glycemic index (GI), and to examine if ethnic groups that showed the largest glycemic response to sucrose would benefit the most when it is replaced with isomaltulose. Forty healthy participants (10 Chinese; 10 Malays; 10 Caucasians; and 10 Indians) consumed beverages containing 50 g of sucrose or isomaltulose on two separate occasions using a randomized crossover design. Capillary blood glucose was measured in a fasted state and at 15, 30, 45, 60, 90, and 120 min after beverage ingestion. Glycemic response to sucrose was significantly higher in Malays compared to Caucasians (*p* = 0.041), but did not differ between Caucasians vs. Chinese (*p* = 0.145) or vs. Indians (*p* = 0.661). When sucrose was replaced with isomaltulose, glycemic responses were significantly reduced in all ethnic groups, with the largest reduction in glycemic response being observed in Malays. Malays, who had the greatest glycemic response to sucrose, also showed the greatest improvement in glycemic response when sucrose was replaced with isomaltulose. This implies that Malays who are more susceptible to type 2 diabetes mellitus may benefit from strategies that replace high GI carbohydrate with lower GI alternatives to assist in glycemic control.

## 1. Introduction

Ethnicity has been widely recognized as a factor that influences the pharmacokinetics and pharmacodynamics of drugs. In contrast, the role of ethnicity in influencing the response to food and food ingredients has only recently been recognized. Diabetes management via lifestyle modifications—especially through diet—is important to improve blood glucose control and to reduce the risk of developing other metabolic complications like cardiovascular disease, nephropathy, neuropathy, and retinopathy [1,2]. In Singapore, Malays and Indians have the highest prevalence of diabetes, at 16.6% and 17.2% respectively, compared to Chinese (9.7%) [3]. A unique metabolic feature of these South Asians is that identical carbohydrate loads elicit postprandial glucose peaks that are 2–3 times larger than Caucasians [4,5,6]. These metabolic differences noted in Asians make it even more important to understand how dietary carbohydrates should be targeted to optimize blood glucose regulation among Asians. 

The prevalence of type 2 diabetes mellitus has been steadily increasing in the past decades. Asian populations (such as Indians, Chinese, Malays, and Thais) were reported to have higher risks in the development of obesity and metabolic syndrome including insulin resistance, type 2 diabetes mellitus, and cardiovascular diseases than Caucasians [4,7]. Asians have greater genetic predisposition to diabetes [8], which is amplified by the greater consumption of high carbohydrate diets and lower physical activity in this region [9,10].

Foods that rapidly increase postprandial blood glucose are referred to as foods with high glycemic index (GI). The large increment in glucose concentration induced by high GI foods often exaggerates the body’s normal anabolic responses [11], which facilitates the overproduction of insulin and eventually results in pancreatic beta cell failure, causing type 2 diabetes mellitus [12].

A series of food ingredients have been identified in recent years to reduce the high glycemic response (GR) induced by high GI foods. These ingredients include beta-glucan, inulin, polyphenols, and isomaltulose [13,14]. Dietary sucrose—commonly known as table sugar—is a disaccharide that has recently come under scientific scrutiny, as it is purported to be a “high GI” food. In fact, sucrose is a medium GI food of 65. Isomaltulose is also a disaccharide, but it is structurally different from sucrose because the saccharides are linked by a α1-6-glycosidic bond between glucose and fructose, instead of the α1-2-glycosidic linkages found in sucrose [15,16]. Due to the difference in the glycosidic linkages, isomaltulose is slowly—albeit completely—digested and absorbed by the small intestine [13,17]. Due to the lower digestion and absorption rates, isomaltulose has a low GI of 32.

In this experiment, we aimed to confirm if the differential GR between Caucasians and Asians reported by previous studies [4,5,6] holds when a comparison between two disaccharides—namely sucrose and isomaltulose—was made. We further investigated whether the ethnic groups that showed greater GR to carbohydrates would benefit more when sucrose (GI = 65) was replaced with the lower GI substitute isomaltulose. This was measured by comparing the GR of the same subjects in each ethnic group either given sucrose or isomaltulose. We hypothesized that all Asian ethnic groups in Singapore (Chinese, Malays, and Indians) would show greater GR than Caucasians, and that Asians would also show a sharper decline in GR than Caucasians when a higher GI sucrose was replaced with a lower GI isomaltulose. 

## 2. Materials and Methods

### 2.1. Participants

The inclusion criteria were healthy males and females aged between 21 and 40 years, fasting blood glucose of <6.0 mmol/L, BMI between 18 and 30 kg/m^2^, both parents and grandparents were of the same ethnicity, no food intolerances or allergies to isomaltulose or sucrose, no metabolic, gastrointestinal, or chronic diseases, not taking insulin or drugs known to affect glucose metabolism and body fat distribution, no major medical event requiring hospitalization within the preceding 3 months, and not pregnant. In order to avoid type 2 error in GR studies, a sample size of 6–10 participants was recommended [18,19]. In addition, based on the previous study conducted by Dickinson, Colagiuri, Faramus, Petocz, and Brand-Miller [4], a total of 40 participants (10 Chinese, 10 Malays, 10 Caucasians, and 10 Indians) were targeted. Participants were recruited from the National University of Singapore campus via study flyers, and the ethnicity of participants was self-reported as described in the inclusion criterion above. To increase the recruitment of Caucasians, invitation emails were also sent to expatriates working in the various research institutions. Participants gave their written consent prior to study commencement. The study was conducted in accordance with the Declaration of Helsinki, and ethical approval was obtained from the National Healthcare Group Domain Specific Review Board (DSRB), Singapore (Reference no. 2014/01008). This trial was registered with ClinicalTrials.gov (Identifier no. NCT03020485).

### 2.2. Study Protocol

All participants were asked to attend two non-consecutive test days where they were given test beverages containing 50 g of isomaltulose (low GI) or sucrose (medium GI) carbohydrates in a random order (Randomizer.org). Participants arrived in the morning after a 10–12 h overnight fast. Blood pressure and basic anthropometric measurements were taken during the first visit. After a ten-minute rest, two fasting blood samples were obtained 5 min apart to determine the baseline blood glucose. An additional fasting blood sample was taken if the first two baseline readings were more than 0.2 mmol/L apart. Participants were instructed to consume either 50 g of sucrose (GI = 65) or 50 g of isomaltulose (GI = 32) powder dissolved in 250 mL water within 5 min. Further blood samples were taken at 15, 30, 45, 60, 90, and 120 min. The first two drops of blood were discarded, and the next drop was used for testing. Blood glucose concentrations were measured using the HemoCue 201+Glucose analyzer (HemoCue, Ängelholm, Sweden) [20], which has a CV of 1.8% [21].

### 2.3. Statistical Analysis

The increment in GR was calculated as the difference between fasting and postprandial blood glucose readings. The temporal incremental GR over 120 min was compared using repeated-measures ANOVA with post hoc Bonferroni corrections. The Total GR over 120 min was also calculated as the incremental area under the curve (iAUC) using the trapezoidal rule that ignores the areas below the baseline. Values are reported as means and standard deviations. One-way ANOVA was used to compare the means of baseline characteristics as well as the iAUC of sucrose and isomaltulose between four ethnic groups. Fisher's least significant difference test was also performed. Effect size using Cohen’s d was calculated based on the differences in iAUC between ethnic groups, with Caucasians used as the comparator. Statistical analyses were performed using SPSS version 23.0 (IBM Corp, Armonk, New York, NY, USA) and two-tailed statistical significance was set at *p* < 0.05.

## 3. Results

**Figure 1:**
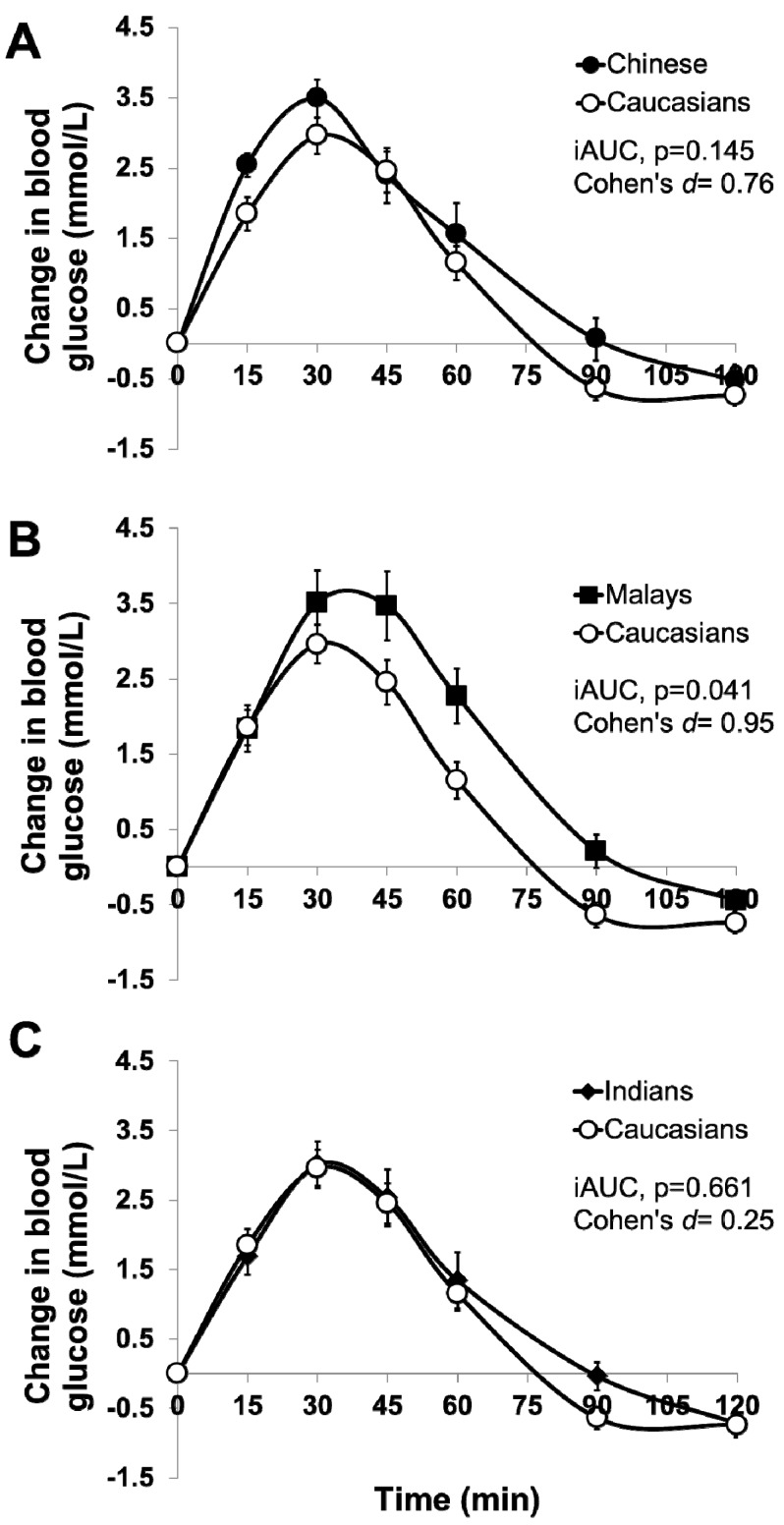
Temporal curves of the blood glucose response to 50 g of sucrose beverage in Caucasians vs. (**A**) Chinese, (**B**) Malays, and (**C**) Indians. Values are means ± Standard Error of Mean. The open circles represent Caucasians, closed circles Chinese, closed squares Malays, and closed diamond Indians. iAUC: incremental area under the curve.

Ten participants from each ethnic group completed the study. All ethnic groups had equal numbers of males and females, except for Indians (seven males and three females). Participants’ demographic characteristics are presented in Table 1. All baseline anthropometric characteristics except for height (*p* = 0.047) did not differ between ethnic groups. After consuming the sucrose beverage, blood glucose increased significantly over 120 min in all four ethnic groups, but postprandial blood glucose changes were not significantly different in all ethnic groups (Time X Ethnic effects, *p* = 0.152) (Figure 1A–C). When expressed as iAUC, the GR of sucrose was significantly different between Caucasians and Malays (*p* = 0.041, Cohen’s d = 0.95), but it did not differ between Caucasians and Chinese (*p* = 0.145, Cohen’s d = 0.76), or between Caucasians and Indians (*p* = 0.661, Cohen’s d = 0.25) (one-way ANOVA, *p* < 0.05 Table 2).

**Figure 2:**
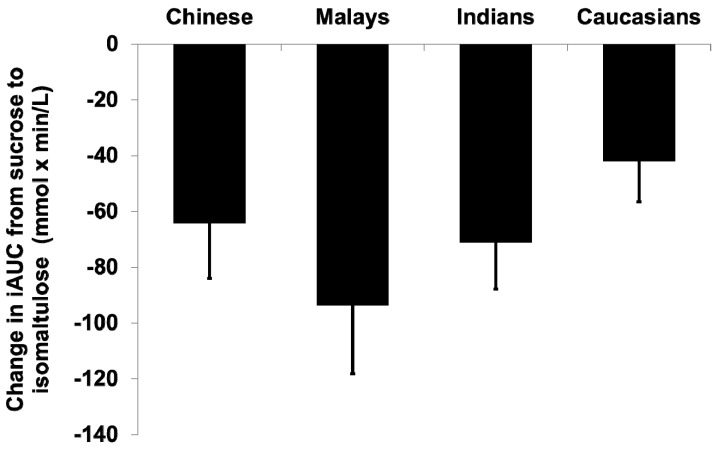
Changes in glycemic response iAUC based on ethnic groups when sucrose was replaced with isomaltulose.

Postprandial glucose following isomaltulose consumption peaked at 45 min compared to the 30 min observed in the sucrose treatment. The substitution of sucrose with isomaltulose reduced within-subject’s GR iAUC over 120 min (iAUC sucrose = 160.1 ± 67.5 mmol/L × min vs. iAUC isomaltulose = 92.4 ± 42.6 mmol/L × min) significantly within (*p* < 0.001), but not between ethnic groups (*p* = 0.241) (Table 2). Although no overall significant difference was observed in the decrease in iAUC after the substitution of sucrose with isomaltulose (one-way ANOVA, *p* = 0.067), the Malays group had the largest decrease in GR iAUC (−93.6 ± 77.9 mmol/L × min), whereas Caucasians had the smallest (−42.1 ± 45.7 mmol/L × min) (one-way ANOVA, *p* < 0.05 Table 2 and Figure 2).

## 4. Discussion

The primary aim of this study was to examine if Asians’ GR to carbohydrate is higher than Caucasians, as previously reported [4,5,6]. In our study, no significant differences in GR were observed between all Asian ethnic groups (Chinese, Malays, and Indians) after the ingestion of 50 g sucrose. However, all Asian ethnic groups had higher GR compared to Caucasians (albeit not statistically significant) in the order of Malays > Chinese > Indians > Caucasians, except for Malays vs. Caucasians (*p* = 0.041). While not completely in line with previous studies [4,5,6,20,22], our findings nevertheless indicate that Asians had a higher GR than Caucasians when a similar amount of carbohydrates were ingested. Since our study recruited populations with comparable age and BMI to previous studies (i.e., relatively young and lean), smaller differences between Asians and Caucasians observed in our study were more likely to be explained by: (1) relatively smaller dose of carbohydrate (CHO) used in our study (50 g vs. 75 g of CHO used in previous study) [4], and (2) sucrose was selected in our study while glucose was used in the previous study. Glucose has a GI of 100, while sucrose has a medium GI of 65. Together with a larger dose of 75 g glucose used in other studies, the high GI glucose was expected to induce greater differential GR between Caucasians and Asians than sucrose used in our present study. Due to the smaller differences in GR between ethnic groups (smaller effect sizes, Cohen’s d, as shown in Figure 1), failure to detect statistical significance in our study was largely attributed to smaller sample size. Based on our retrospective sample size calculation, increasing the sample size for Chinese to (*n* = 29) would allow us to detect statistical significance relative to Caucasians.

Compared with Caucasians, the GR to sucrose was significantly higher in Malays. It was an important observation, given that both ethnic groups had comparable BMI (23–24 kg/m^2^) and fasting blood glucose (4.5–4.8 mmol/L) within the healthy range. This highlights the increased risk for impaired glucose tolerance among healthy Malays, even at a smaller dose (50 g) of the medium-GI carbohydrate sucrose. An important clinically-relevant observation was that in Malays (who had the highest GR among all ethnic groups), the substitution of sucrose with isomaltulose produced the largest decline in GR. All other Asian ethnic groups also showed greater decreases in GR relative to Caucasians when sucrose was replaced by isomaltulose. The lack of statistical significance in other Asian ethnicities was predominantly due to sample size limitations, but the clinical importance of the observation in Malays is to be noted. Nathan et al. [23] reported that an average 1.6 mmol/L decrease in acute postprandial blood glucose could translate into 1% decrease in glycated hemoglobin (HbA1c) over longer terms. Our findings suggest that Asians may benefit more from the inclusion of lower GI ingredients into foods than Caucasians. This is especially important in the context of Asian cuisines where carbohydrates such as rice, noodles, and bread are major staple foods and are consumed in large quantities. Based on the National Nutrition survey in Singapore, the average daily consumption of carbohydrates in adults is 337.4 g [24]. The inclusion of lower GI ingredients such as isomaltulose into meals will potentially decrease the overall postprandial GR in Asians, especially in Malays. Although the current study was carried out in healthy participants, the extrapolations of these results in individuals with diabetes and pre-diabetes merits further investigation. 

## 5. Conclusions

In conclusion, all Asian populations—especially Malays—were shown to have significantly higher GR than Caucasians to both sugars. These results indicate that the impact and relevance of replacing sucrose with isomaltulose may have a significant influence on glycemic control in Malays who have greater susceptibility to type 2 diabetes mellitus.

## Figures and Tables

**Table 1:** Anthropometric characteristics of the study participants by ethnicity.

Characteristics	Chinese (*n* = 10)	Malays (*n* = 10)	Indians (*n* = 10)	Caucasians (*n* = 10)	*p*-Value
Age (years)	24.2 (3.9)	25.8 (4.3)	23.6 (2.1)	27.5 (5.2)	0.150
Weight (kg)	59.8 (8.2)	61.3 (13.7)	67.1 (9.3)	70.6 (10.9)	0.104
Height (cm)	1.67 (0.1)	1.62 (0.1)	1.67 (0.1)	1.73 (0.1)	0.047 *
Body mass index (kg/m^2^)	21.3 (1.3)	23.0 (3.2)	24.1 (3.0)	23.6 (2.7)	0.105
Systolic blood pressure (mmHg)	107.6 (7.7)	110.4 (10.1)	110.7 (7.0)	117.6 (8.2)	0.071
Diastolic blood pressure (mmHg)	71.4 (5.5)	70.0 (12.1)	69.4 (7.2)	70.9 (6.6)	0.946
Mean fasting blood glucose (mmol/L)	4.7 (0.3)	4.5 (0.4)	4.4 (0.5)	4.8 (0.4)	0.181
Waist circumference (cm)	71.4 (5.8)	72.2 (10.1)	76.1 (7.9)	76.9 (8.8)	0.366
Hip circumference (cm)	93.6 (4.7)	95.8 (5.2)	98.7 (6.5)	97.2 (7.7)	0.305

All values are means (Standard Deviations). * Statistically different, one-way ANOVA. *p* < 0.05.

**Table 2:** Glycemic responses to sucrose and isomaltulose as incremental AUC (iAUC).

Ethnicity	iAUC Glucose (mmol × min/L)		Difference	*p*-Value
	Sucrose (50 g)	Isomaltulose (50 g)		
All (*n* = 40)	160.1 (67.5)	92.4 (42.6)	−67.7 (61.5)	<0.001
Chinese (*n* = 10)	174.1 (69.6)	109.9 (27.6)	−64.1 (62.8)	0.010
Malays (*n* = 10)	192.4 (82.6) ^a^	98.8 (52.0)	−93.6 (77.9)	0.004
Indians (*n* = 10)	143.4 (60.8)	72.3 (31.4)	−71.2 (52.9)	0.002
Caucasians (*n* = 10)	130.5 (41.3) ^a^	88.4 (50.7)	−42.1 (45.7)	0.017

All values are means (Standard Deviations). ^a^ Mean values were significantly different between Malays and Caucasians (*p* = 0.041).
